# Investigation of spin wave dynamics in Au/CoFeB/Au multilayers with perpendicular magnetic anisotropy

**DOI:** 10.1038/s41598-023-49859-8

**Published:** 2023-12-15

**Authors:** S. Janardhanan, S. Mielcarek, H. Głowiński, M. Kowacz, P. Kuświk, M. Krawczyk, A. Trzaskowska

**Affiliations:** 1 ISQI, Faculty of Physics, Adam Mickiewicz University, Poznan, Poland; 2 Institute of Molecular Physics, Polish Academy of Science, Poznan, Poland

**Keywords:** Ferromagnetism, Magnetic properties and materials, Surfaces, interfaces and thin films

## Abstract

We have carried out an experimental investigation of the spin-wave dynamics in the Au/CoFeB/Au multilayer consisting of a ferromagnetic film with thicknesses of 0.8, 0.9 and 1.0 nm. We employed the Brillouin light scattering spectroscopy to measure the frequency of the spin waves in dependence on the wave vector. Additionally, we characterized the samples by ferromagnetic resonance measurements. We found that the considered samples exhibit perpendicular magnetic anisotropy with low damping, indicating small pumping effects. Furthermore, we found a nonreciprocal dispersion relation pointing at a non-negligible Dzyaloshinskii–Moriya interaction. These results make the Au/CoFeB/Au multilayer a compelling subject for further analysis and as a potential material for future applications within magnonics.

## Introduction

Currently, there is an increasing demand for mobile and stationary devices that support wireless communication. These devices are part of the Internet of Things ecosystem. This progress requires small and energetically efficient devices at permanently increased frequencies and transfer rates^1,2^. The field of spin waves (SWs), and so magnonics, is one which has gained a lot of attention over a 10-year horizon in the scientific community and is considered a promising approach to fulfil these requirements. This is because SWs can operate at high frequencies and transfer a spin for a large distance without charge transfer, which minimizes the energy cost of the logic operations^3,4^. To use SWs, we have to know how to control, excite and detect them. Control of the SW dynamics can be achieved by tuning the SWs with the external magnetic field, by material structuration or by affecting the static magnetic configuration. Moreover, magnonic devices are easily reconfigurable that make them favorable for active control, difficult to achieve for other devices from photonics or electronics^5–8^. Therefore, the magnonic systems can fill the gap between ultra-fast photonics and extremely miniaturized electronic systems in order to design energetically efficient logic devices, miniaturized below 100 nm and operating at relatively high frequencies in the range from few to tens of GHz. However, to achieve this goal, it is necessary to research new materials that would enable the propagation of SWs over long distances and dynamic control of their properties.

The Dzyaloshinskii–Moriya interaction (DMI) is an anisotropic exchange interaction governing chiral textures, arising from the interplay between two atomic spins and a neighboring atom with substantial spin–orbit interactions^9^. Interfacial DMI emerges in ultrathin ferromagnetic films (FM), typically in the presence of a heavy metal (HM), within systems lacking inversion symmetry due to strong spin–orbit coupling^10–12^. It serves as a source of nonreciprocity in both frequency and amplitude of SWs, a phenomenon extensively explored in recent years. In this scenario, SWs with the same wave number but propagating in opposite directions exhibit different frequencies^13^. Experimental verification of DMI often involves Brillouin light scattering (BLS), specifically by measuring the discrepancy between the frequencies of Stokes and anti-Stokes signals^14^. Recently, DMI has been actively investigated in various material combinations possessing perpendicular magnetic anisotropy (PMA) owing to their capacity to generate magnetic textures with predetermined chirality^12,15–17^. These properties are relevant for magnonic applications. Nevertheless, the multilayers demonstrating PMA and DMI frequently have substantial damping, restricting their usefulness in magnonics.

We have chosen an ultra-thin CoFeB film within a multilayer structure comprising Si/Ti/Au/CoFeB/Au for our examination. Previous studies with a similar composition, as outlined in Refs.^18,19^, were primarily focused on dispersion analysis and magnetoelastic interactions. Moreover, prior research^20–26^ has demonstrated that CoFeB, when situated between diverse layers such as oxides (e.g., MgO^20,21^), noble metals (like Pt^22^ or Pd^23^), or heavy metals (e.g., Hf, Ta, and W^24–26^), exhibits DMI and PMA. Despite this, CoFeB between Au layers has received relatively little attention so far^27^, even though these structures can display significant PMA. Furthermore, film systems based on Au exhibit minimal small spin-pumping effects, which constitute the important contributor to SW damping in thin films in contact with heavy metals. Hence, Au/CoFeB/Au can be considered as a promising candidate for future applications, particularly in magnonics and spintronics, where PMA and low damping are crucial^28,29^.

Our research is grounded in the experimental investigation of SWs in structures with PMA, specifically Ti/Au/CoFeB (0.8 nm, 0.9 nm, 1 nm)/Au deposited on a silicon substrate. Established tools like BLS and vector network analyzer ferromagnetic resonance (VNA-FMR) are employed for SW characterization. From the dispersion relation, we have identified the existence of DMI alongside PMA in this multilayer system. Notably, we observed relatively low damping in both BLS and VNA-FMR measurements. The synergy of PMA and DMI with low damping makes this multilayer system a prospective candidate for applications in magnonic devices and spintronics. We anticipate that these findings will stimulate further research in this field in near future.

## Materials and methods

### The sample

**Figure 1:**
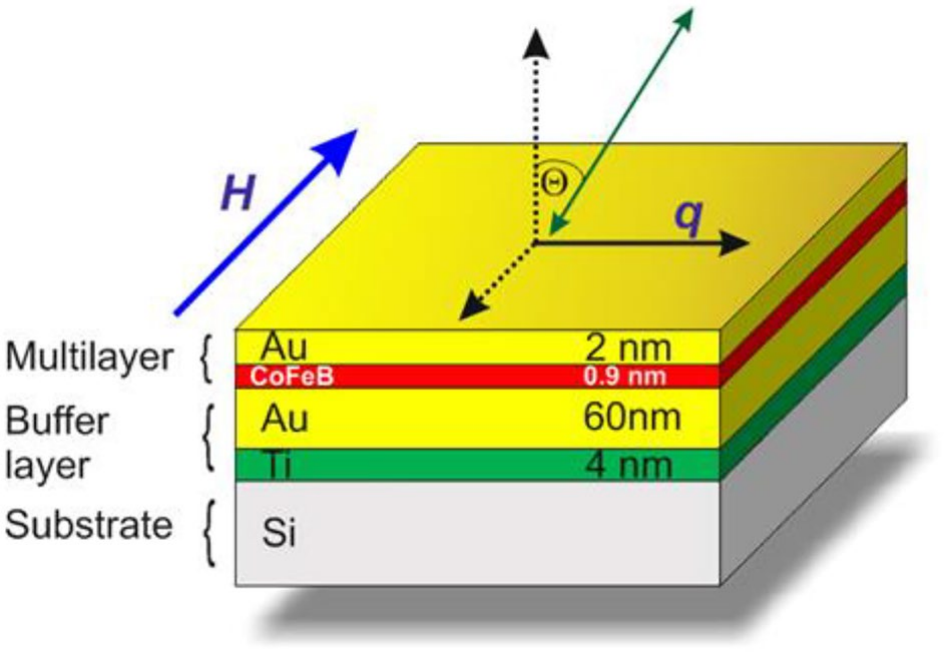
Schematic sketch of one of the considered PMA systems Si/Ti(4 nm)/Au(60 nm)/CoFeB (0.9 nm)/Au(2 nm). The wave vector (*q*) of SWs is visible on the surface. The magnetic field *H* is applied in the plane of the sample and is perpendicular to the plane of the incident light (defined by normal to the sample and *q* vector). The wave vector of SWs (*q*) is perpendicular to the applied field *H* (i.e., Damon–Eshbach (D–E) geometry). The green arrow represents incident/scattered light.

The Ti(4 nm)/Au(60 nm)/CoFeB(*t*_CoFeB_)/Au(2 nm) sample with* t*_CoFeB_ = 0.8, 0.9, 1.0 nm was deposited onto naturally oxidized (001) silicon substrate using magnetron sputtering in Ar atmosphere at *p*_Ar_ = 1.4 × 10^–3^ mbar. The deposition was performed with base pressure < 2 × 10^–8^ mbar. The dimensions of the sample were 5 × 10 mm^2^. The CoFeB layer was sputtered from a Co_20_Fe_60_B_20_ target, the composition of which was earlier verified by energy dispersive X-ray spectroscopy^27^. The Ti, Au, and CoFeB thicknesses were controlled by deposition time according to the deposition rate determined by profilometer measurements from the calibration sample. An amorphous phase of CoFeB was verified by an x-ray diffractometer in grazing incident configuration^27^. A view sketch of the sample is shown in Fig. 1.

### Experimental setup

Experimental studies were done by using a six-pass tandem Brillouin spectrometer (TFP2-HC, JRS)^30,31^ ensuring the contrast of 10^15^, a single-mode diode-pumped laser was used as the source of light, emitting the second harmonics of the length *λ*_*0*_ = 532 nm^32,33^. Measurements were made in the backscattering geometry with the light polarization *ps* for magnons. The backscattered light was collected by using f/8 optics with a focal length of 150 mm. The BLS allowed direct measurement of SW frequency present in the structure at the particular scattering vector corresponding to the position in the *q*-space. The geometry for SWs used here was Damon–Eshbach (D–E) (Fig. 1), i.e., SW wave vector *q* was perpendicular to the in-plane magnetic field, which saturated the sample. The scattering wave vector *q* was determined by incident light, *q* = (4π/λ_0_)sinƟ, where Ɵ was the angle between incident light and normal to the sample surface (Fig. 1). In our experiments, the wave vector varied between 0.03 × 10^7^ m^−1^ and 2.25 × 10^7^ m^−1^ with a resolution of about 0.01 × 10^7^ m^−1^. Each spectrum was collected for 1-h, and each peak within the spectrum was fitted using a Lorentzian curve. Further details regarding our experimental setup are available in Ref.^34^. Magnetic field measurements were conducted in the range of 2000 Oe to 8000 Oe. All experiments were conducted at room temperature. Standard magnetic characterizations were performed using VNA-FMR and polar magneto-optical Kerr effect (PMOKE). Hysteresis loops were recorded utilizing PMOKE with a laser wavelength of 650 nm and a beam diameter of 0.3 mm focused on the sample surface at room temperature. Electromagnets were employed to generate the magnetic field. Samples were positioned on a table equipped with a stepper motor, facilitating automated sample positioning relative to the light spot. This setup enables the realization of magnetization processes along the chosen direction of the sample. Magnetometer measurements enable the tracking of the magnetization process, providing essential values for numerous magnetic parameters.

## Results

The magnetic behavior of the CoFeB thin layer is significantly influenced by interactions at the interface between adjacent layers^35^. Previous findings for similar samples indicate that when the CoFeB film thickness falls within the range of 0.65 nm to 1.0 nm, the layer remains magnetically continuous, exhibiting strong PMA^27^. To assess the dynamic magnetic parameters of the sample, we examined its magnetic field dependence using VNA-FMR and BLS with normal incidence of light (*θ* = 0.7°, *q* ≈ 0), corresponding to a uniform precession mode.

**Figure 2:**
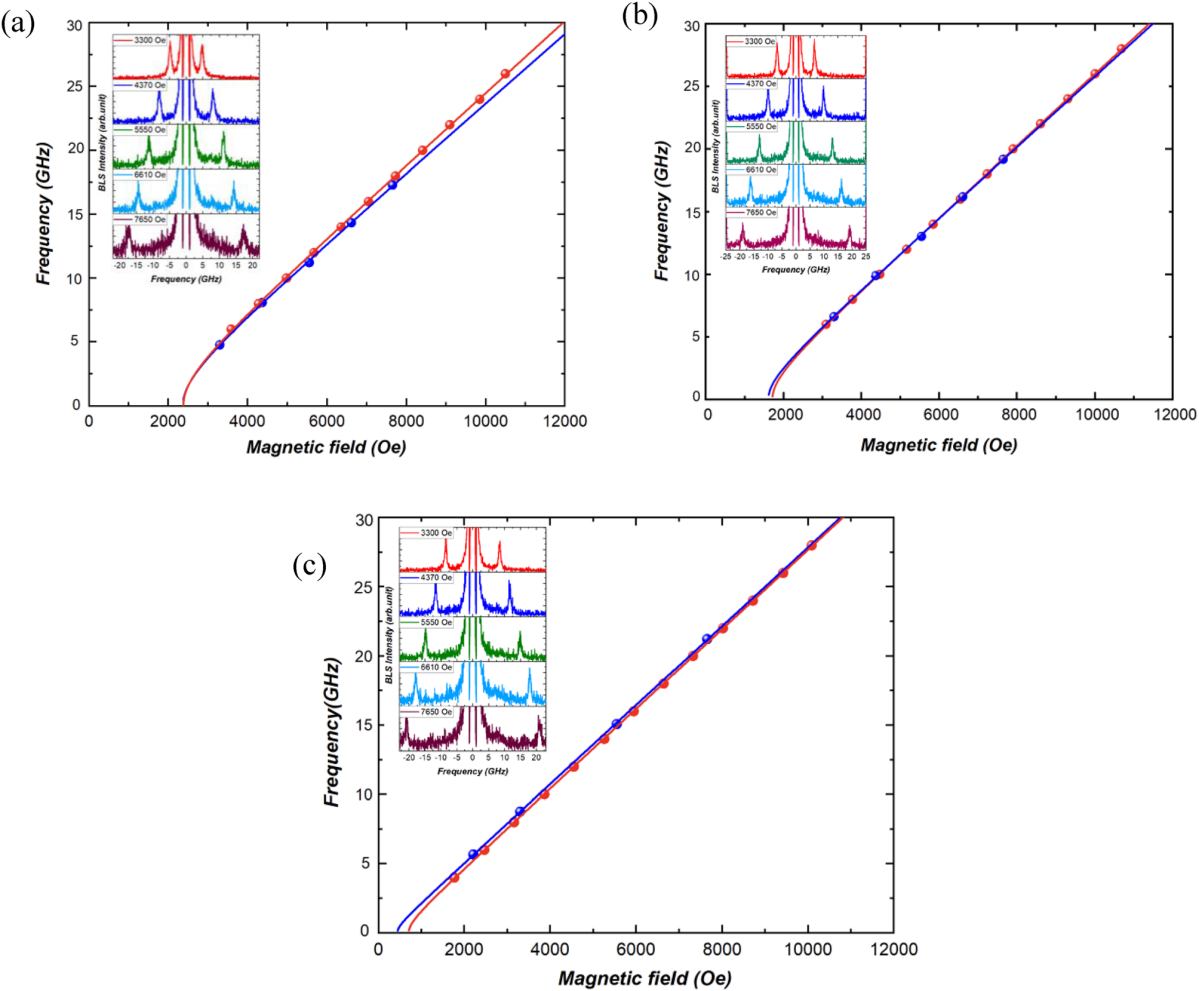
Results of the magnetic field dependence study of the multilayer with (**a**) *t*_CoFeB_ = 0.8 nm, (**b**) *t*_CoFeB_ = 0.9 nm, and (**c**) *t*_CoFeB_ = 1 nm, showing a comparison between VNA-FMR spectra (red points) and BLS spectra (blue points). The experimental points are fitted using Kittel’s equation (blue and red lines for BLS and VNA-FMR respectively). Inset: Brillouin light scattering spectra measured under different magnetic field values for CoFeB film of (**a**) 0.8 nm, (**b**) 9 nm, and (**c**) 1 nm thickness.

Figure 2 (blue points) displays BLS spectra of a Si/Ti(4 nm)/Au(60 nm)/CoFeB(*t*_*CoFeB*_)/Au(2 nm) under various magnetic field values (*H*). Both Stokes and anti-Stokes peaks, visible in the presented spectra (insets in Fig. 2) and corresponding to SW propagating in opposite directions, were simultaneously observed with comparable intensity. The frequency of both Stokes and anti-Stokes peaks increases with the magnetic field, confirming their SW origin^4^. Figure 2 also illustrates a comparison between the results obtained from BLS and VNA-FMR (red points), showing good agreement between the two measurement techniques.

Using the Kittel formula^27^:

1 $$f=\frac{\gamma }{2\pi }\sqrt{H\left(H+4\pi {M}_{{\text{eff}}}\right)} ,$$

where *H* is the magnetic field, $\gamma =\frac{g{\mu }_{B}}{\hslash }$—is the gyromagnetic ratio, *ћ*—is the reduced Planck constant, *µ*_B_—is the Bohr magneton, we extracted the effective magnetization (*4* $\pi$ *M*_eff_) and Lande factor (*g*) characteristic of the studied sample from both FMR and BLS results. They are collected in Table 1. The effective magnetization *4* $\pi$ *M*_eff_ describes the effective anisotropy (*4* $\pi$ *M*_eff_ = ${2K}_{{\text{eff}}}/{M}_{{\text{s}}}$). The negative *M*_eff_, extracted from measurements, means that the magnetic easy axis is perpendicular to the film with PMA prevailing shape anisotropy^36^.

**Table 1:** Lande factor *g*, effective magnetization *M*_*eff*_ and effective damping constant *α* from VNA-FMR and BLS measurement for 0.8, 0.9, and 1.0 nm samples.

Parameters	*t*_CoFeB_ = 0.8 nm		*t*_CoFeB_ = 0.9 nm		*t*_CoFeB_ = 1 nm	
	VNA-FMR	BLS	VNA-FMR	BLS	VNA-FMR	BLS
*g*	2.01 ± 0.01	2.003 ± 0.01	2.039 ± 0.003	2.011 ± 0.01	2.068 ± 0.003	2.04 ± 0.01
*4* $\pi$ *M*_*eff*_ (Oe)	− 2412 ± 37	− 2375 ± 34	− 1652 ± 20	− 1608 ± 50	− 308 ± 12	− 427 ± 65
$\alpha$	0.0087 ± 0.0015	0.0084 ± 0.0012	0.0085 ± 0.0015	0.011 ± 0.0008	0.0124 ± 0.0008	0.010 ± 0.003
*ΔH*_*0*_ (Oe)	279 ± 20		245 ± 20		163 ± 10	

**Figure 3:**
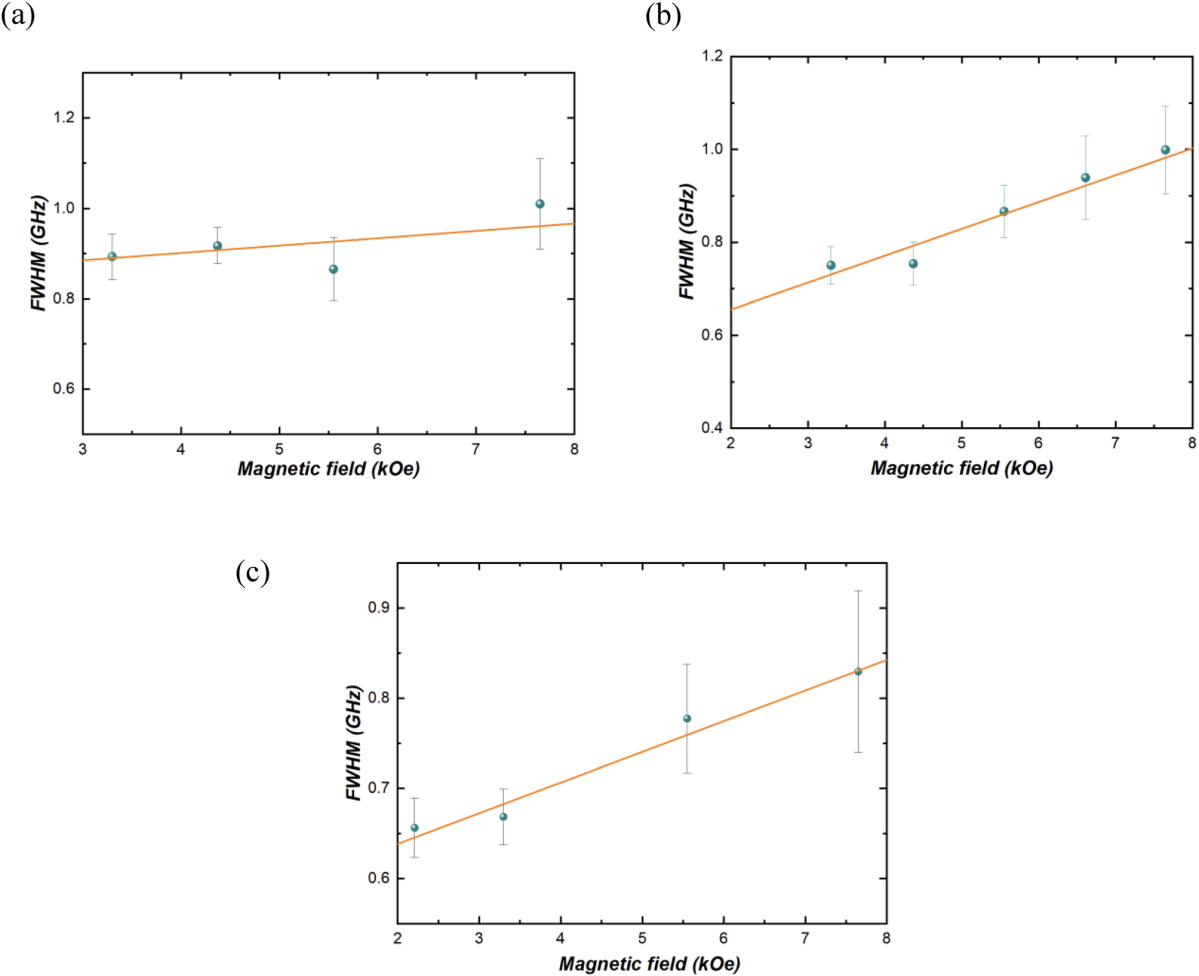
Full width at half maximum (FWHM) plotted against the magnetic field (*H*) obtained from BLS measurements shown in Fig. 1, for (**a**) 0.8 nm, (**b**) 0.9 nm, and (**c**) 1 nm thick CoFeB samples.

From the BLS and VNA-FMR measurements, we can also extract the linewidth taken as the full width at half maximum (FWHM) in frequency and magnetic field domain, respectively. The extraction of damping from BLS measurements is not often used but has been previously employed by other research groups, with results consistent with other measurement methods, see Refs.^37,38^. This validates the utility of BLS for estimating effective damping. The results from BLS data are shown in Fig. 3. It varies linearly with the magnetic field, and can be described by:

2 $$FWHM=\left(2\alpha \gamma /\pi \right)H+\delta {f}_{0},$$

where, α is an intrinsic effective SW damping, and δ*f*_0_ is extrinsic linewidth unrelated to *H*, which comes mainly from the instrument’s built-in linewidth from the interferometer and sample inhomogeneity^22,37^. By utilizing Eq. (2), we derive *α* values for the analyzed samples, as presented in Table 1. The linear fits yielded correlation coefficients of 0.9, 0.95, and 0.96, respectively.

**Figure 4:**
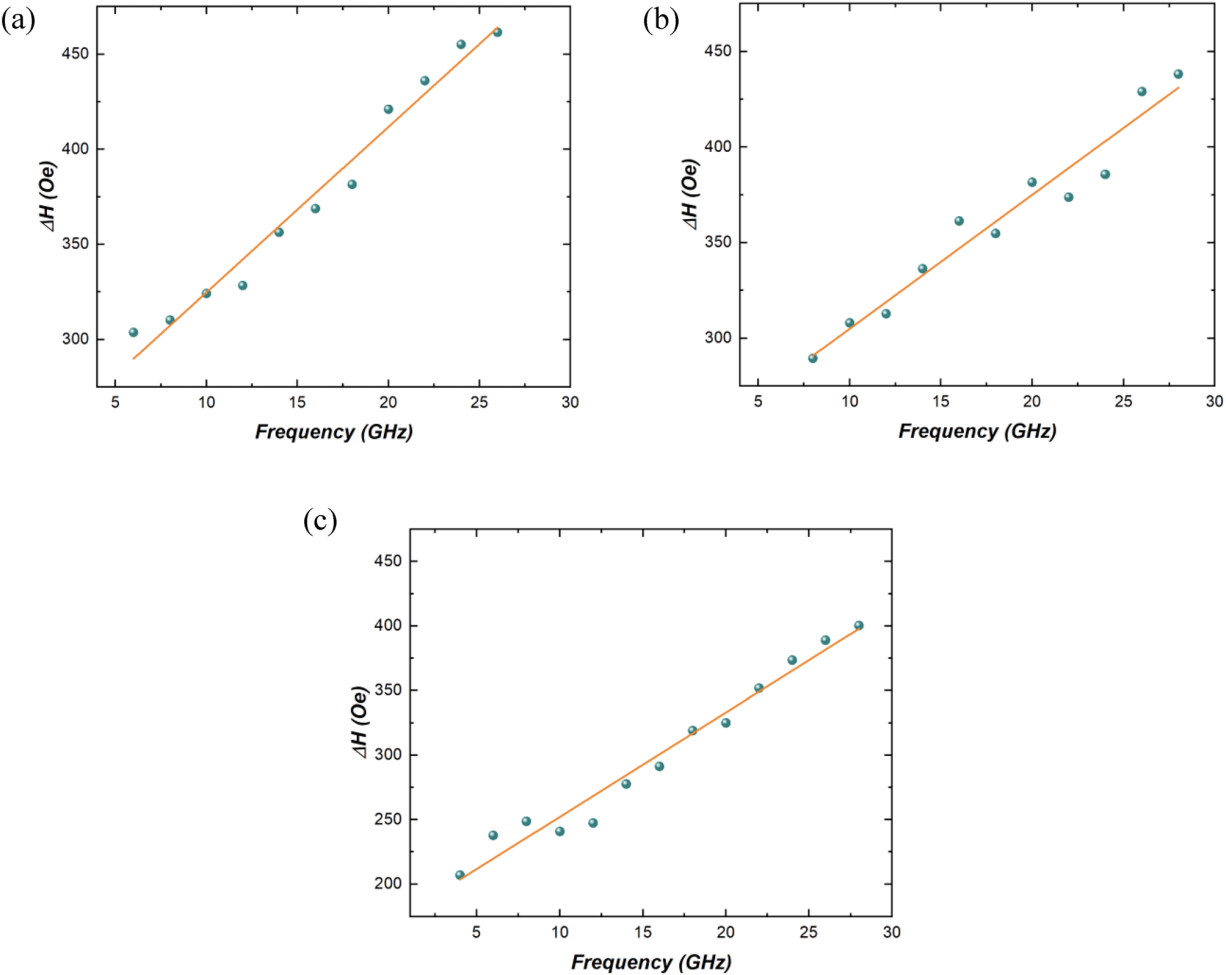
Resonance linewidth (*ΔH*) dependence on frequency *(f)* obtained from VNA-FMR measurements for (**a**) 0.8 nm, (**b**) 0.9 nm, and (**c**) 1 nm thick CoFeB samples.

In the same way, from VNA-FMR, we determined the resonance linewidth (Δ*H*) of the absorption peak, see Fig. 4, which also reflects both intrinsic effective damping α and inhomogeneous broadening (Δ*H*_0_). According to the Landau–Lifshitz–Gilbert equation Δ*H* shows the linear dependence on the *f*^39,40^ and can be expressed by:

3 $$\Delta H=\left(4\pi \alpha /\gamma \right)f+\Delta {H}_{0}.$$

By fitting the experimental data with Eq. (3) we also obtained: $\alpha$ as well as $\Delta {H}_{0}$ (Table 1).

Hence, the effective damping constant *α* obtained through BLS analysis (Fig. 3) concurs with the results obtained using VNA-FMR (Fig. 4), as presented in Table 1. These findings are in alignment with the values previously reported in the literature^27^.

In the context of multilayers based on noble metals, the damping parameter extracted from measurements can stem from various sources, including intrinsic damping associated with the loss of SW energy to the bulk lattice, spin pumping to gold (Au), as well as contributions from two-magnon scattering or non-local damping^41,42^. In our study, we utilized double layers of Au with different thicknesses on the lower and upper sides of CoFeB. Given that the gold buffer layer is 60 nm thick, exceeding half the spin diffusion length of gold (35 nm at room temperature)^43,44^, the system can be considered as an effective sink^27^. Conversely, the contribution from the cap layer can be disregarded due to its minimal thickness. Unexpectedly, we observed an unusual dependence of the effective attenuation constant on the ferromagnetic film thickness, i.e. a slight increase of *α* with *t*_CoFeB_. The reason for this is not yet clear, but it could be a non-local attenuation, as suggested in the recent study^42^. Nevertheless, this effect requires further investigations, which are beyond the scope of this paper. Despite this, the obtained values of the effective damping parameter (*α*) from both VNA-FMR and BLS measurements align well with values reported in the literature^27,37^.

**Figure 5:**
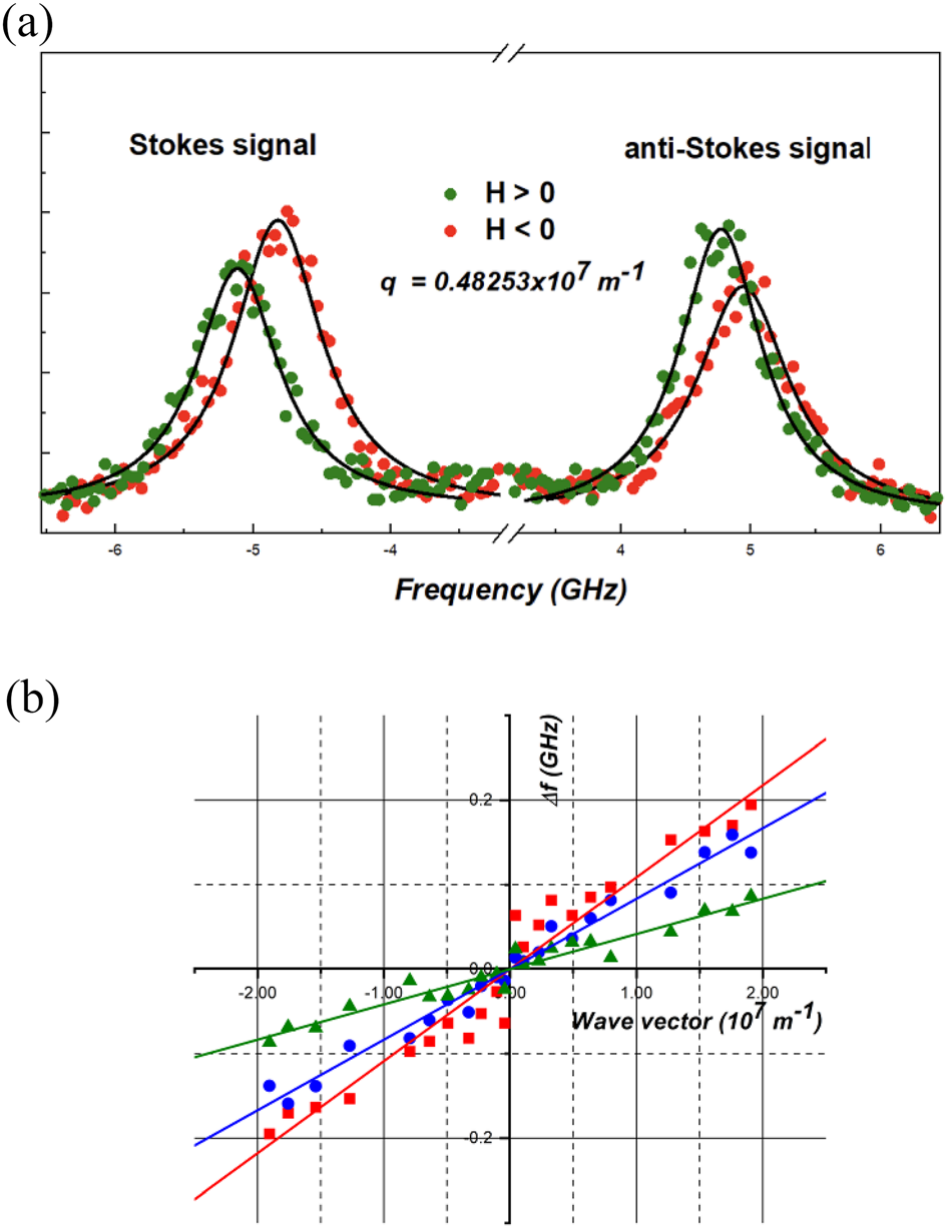
(**a**) Stokes and anti-Stokes BLS signals of the sample with 0.9 nm CoFeB thickness fitted with the Lorentz curve (black lines) under a positively oriented (green) magnetic field (+ 2400 Oe) and negatively oriented (red) magnetic field (− 2400 Oe). (**b**) Frequency difference between the Stokes and the anti-Stokes peaks measured (points) and their linear fitting as a function of the wave vector for samples with the thickness of CoFeB 0.8 nm (red), 0.9 nm (blue) and 1 nm (green) under 2400 Oe.

Application of a magnetic field in two opposite directions reveals differences in Stokes and anti-Stokes peaks in terms of GHz, as depicted in Fig. 5a. The discernible frequency disparity underscores the presence of DMI in this case (Fig. 5b). For SWs propagating perpendicularly to the magnetization in in-plane saturated ultrathin films, the influence of interfacial DMI on the dispersion relation is characterized by a linear frequency shift of SWs propagating at opposite wave vectors *q*^45,46^. Hence, the frequency shift between SWs with opposite wave vectors:

4 $$\Delta f \left(q\right)= \frac{\left|f\left(-q\right)-f\left(q\right)\right|}{2}= \frac{\gamma }{\pi {M}_{S}}Dq,$$

is used to extract the strength of the interfacial DMI (*D*), where *M*_S_ denotes saturation magnetization.

**Figure 6:**
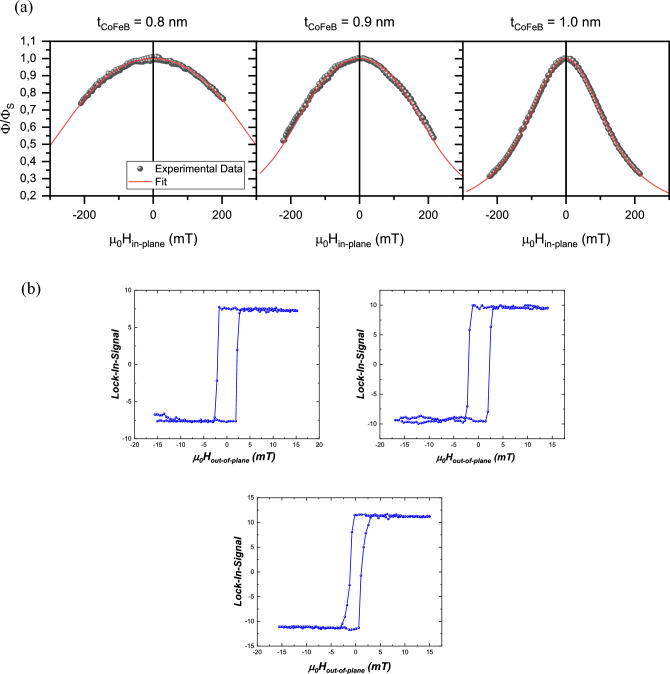
(**a**) PMOKE signal under an in-plane magnetic field and constant out-of-plane magnetic field for Au/CoFeB/Au with different *t*_CoFeB._ The red line shows the best fit for the Stoner–Wohlfarth model. (**b**) Hysteresis loop of the samples with CoFeB thickness 0.8 nm 0.9 nm and 1 nm respectively obtained from the PMOKE.

According to the analytical model, we obtained *D* for samples with different CoFeB thicknesses (Fig. 6b). The highest *D* value was found for *t*_CoFeB_ = 0.8 nm (− 0.101 mJ/m^2^), and the values decrease for the thicker CoFeB layer* (*for *t*_CoFeB_ = 0.9 nm* D* =  − 0.0776 mJ/m^2^ and for *t*_CoFeB_ = 1 nm* D* =  − 0.038 mJ/m^2^)*.* Nevertheless, the DMI sign is clearly preserved for all samples and we can conclude that the DMI is present on CoFeB surrounded by Au layers. This may be quite surprising considering the symmetrical surroundings of the CoFeB layer and recent results, which show that the Au in contact with CoFeB gives the opposite DMI sign from Fe and Co atoms^37^, which in total might give a rather low DMI. The DMI values obtained will be further discussed in relation to the literature in the “Discussion” section.

The effective anisotropy constant (*K*_eff_) was determined using two independent techniques: VNA-FMR and PMOKE. Assuming that the magnetization ${M}_{s}$ is 1150 kA/m we can calculate from VNA FMR the effective anisotropy according to the formula ${K}_{{\text{eff}}}=2\pi {M}_{{\text{eff}}}{M}_{s}$. The obtained values are presented in Table 2. To validate this result, we also performed the PMOKE measurements, which allow us to follow magnetization tilting (by registering the perpendicular component of the magnetization ϕ) under an in-plane magnetic field (*H*_*in-plane*_) and constant out-of-plane magnetic field, which ensures a single domain state. This tilting can be distinguished from the PMOKE signal *ϕ/ϕs* (where *ϕs* is the PMOKE signal in saturation) (Fig. 6), which then can be fitted to the Stoner–Wohlfarth model^40^ (red lines) to extract the *K*_eff_ values for each CoFeB thicknesses (Table 2).

**Table 2:** The *K*_eff_ for the CoFeB layer of considered thicknesses determined from two independent techniques, VNA-FMR and PMOKE.

*t*_CoFeB_ (nm)	*K*_eff_ (MJ/m^3^) VNA-FMR	*K*_eff_ (MJ/m^3^) PMOKE
0.8	− 0.137	− 0.14
0.9	− 0.094	− 0.09
1.0	− 0.018	− 0.03

The values obtained from the PMOKE and VNA-FMR measurements (Table 2) are close to each other and are negative. This confirms that all the investigated samples have PMA, however, the sample with *t*_CoFeB_ = 1 nm is very close to the spin reorientation thickness. Note that both the spin reorientation thickness and the obtained anisotropy values are in good agreement with the results obtained for the Au/CoFeB-wedge/Au investigated by PMOKE above spin reorientation transition^27^. The hysteresis loops of the three samples (Fig. 6b) demonstrate also that the CoFeB layer maintains magnetic continuity and exhibits perpendicular magnetic anisotropy (PMA), as evidenced by its square hysteresis loop.

## Discussion

As previously mentioned, the Au/CoFeB/Au system displays minimal spin pumping effects compared to other noble metals, positioning it as a promising candidate for applications requiring both PMA and low damping characteristics^44^. In this study, we utilized ultra-thin CoFeB with inherent PMA. The observed asymmetry in the dispersion relation cannot be solely attributed to asymmetric surface pinning due to the thin film thickness, as numerical calculations suggest. Instead, it predominantly stems from interfacial DMI. The evident non-reciprocity in the dispersion relation and the unique propagation of DE SWs in multilayer film structures with spin–orbit interactions and broken inversion symmetry^10^ are noteworthy. In our case, where D < 0, it favors left-handed chirality, potentially influencing magnetic phases, configurations, and phenomena such as skyrmions and domain walls. The observed sign reversal in DMI is linked to the electronic band structure, specifically the 5d band occupancy of heavy metals. Materials experiencing a transition in the 5d band from less than half-filled to more than half-filled can undergo a sign reversal in DMI. This alteration in electronic configuration influences the interaction of electrons with the crystal lattice, leading to a change in the DMI sign. Samples with Ta and W underlayers exhibit D > 0 with a preference for right-handed magnetic chirality, while those with Pt and Au underlayers have D < 0, favoring left-handed magnetic chirality^37^. Overall, the sign reversal in the DMI constant, connected to the electronic band structure, underscores the intricate interplay between electron spin, crystal lattice, and magnetic interactions in materials, providing insights into the behavior of magnetic structures and spin textures^11,47,48^.

The interfacial DMI value presented in our study appears relatively modest compared to established literature values for CoFeB near heavy/noble metals. This variance can be ascribed to factors such as magnetic material thickness, interaction with adjacent layers, and so on. Remarkably, multilayer systems with more substantial DMI values are identifiable. In the W/CoFeB (0.6–1.5 nm)/MgO configuration, for instance, the DMI spans 0.073–0.88 mJ/m^2^^49^. In the case of the Au/CoFeB (1 nm)/MgO system, the documented DMI magnitude is 0.158 mJ/m^2^^11,37,50^. Different DMI values are evident in HM/CoFeB systems, with HM denoting Ta, W, Pt, Au, Ru, Ir, or IrMn, as outlined in references^46,51^. In such instances, the DMI magnitude varies within the range of 0.036 to 1.13 mJ/m^2^. A similar pattern emerges when considering scenarios where CoFeB is surrounded by oxides like MgO^20^, resulting in a DMI of 1.3 mJ/m^2^. Notably, the unique aspect introduced in our study is the presence of Au as both a buffer and cap layer.

In the context of layered structures, DMI is usually assumed zero if the structure is symmetric, with asymmetry being the prerequisite for nonzero DMI. However, when examining interfaces composed of the same materials, as exemplified by Au/CoFeB and CoFeB/Au in our case, they are not identical. This discrepancy arises from the sequential deposition of gold followed by CoFeB and vice versa, concluding with the deposition of a gold layer on the CoFeB layer. Consequently, these may explain the non-zero DMI value. This effect has already been scrutinized in other studies, e.g., the study^12^ pertaining to Pt/Co/Pt and also explored on various multilayers characterized by nominally symmetric structures, as evidenced in references^29,52^. However, in the existing literature, there are no documented studies on the configuration of CoFeB layers surrounded by gold on both sides. This gap in research was explored in our study.

The table presented in the previous section (Table 1) compiles the results of important parameters extracted from both VNA-FMR and BLS measurements. These measurements were conducted to explore the magnetic and vibrational attributes of the examined samples. The parameters encompass the Lande factor *g*, effective magnetization *M*_*eff*_, and effective damping factor *α*. Each parameter offers insights into specific aspects of the sample’s magnetic and mechanical behaviour. The VNA-FMR and BLS data show consistency with each other here. The slight differences (Table 1) can be interpreted based on measurement uncertainties. These findings serve as a foundation for understanding the sample's behaviour and may contribute to advancements in fields such as materials science, condensed matter physics and magnetic device engineering.

Several factors may contribute to the nonreciprocal dispersion relation of SWs in the DE configuration, aside from DMI itself. Metallization of one side of the ferromagnet can cause asymmetrical screening of the stray magnetic field, resulting in an asymmetric dispersion relation. However, this effect requires a surface character of SWs at large wavenumbers, which is not applicable to our study focused on ultrathin films^53^. Another potential source is asymmetric surface anisotropy, introducing different magnetization pinning on film surfaces^54^. While this was considered theoretically, its contribution to our study is not the primary cause of the observed nonreciprocity due to small CoFeB thicknesses. The presence of a magnetization gradient, as demonstrated in a recent study, may enhance nonreciprocity in the presence of DMI^55,56^. However, given the small thickness of our 0.8–1.0 nm CoFeB films and their limited number of magnetic monolayers, the gradient is likely negligible. In summary, the observed nonreciprocal dispersion relation, linearly dependent on wavenumber in the DE configuration, is reasonably attributed to the presence of DMI itself.

Multilayer systems with PMA based on noble elements such as Pt, and Pd, possess significant contributions to damping from spin pumping effects^22,41,57^. According to our study and Ref.^27^, the contribution of spin pumping to Au is comparatively lower than the damping observed in the CoFeB layer with other noble metals, which makes measured effective damping relatively low. Interestingly, for ultrathin multilayers Ta/CoFeB (1 nm thick)/MgO, the intrinsic damping parameter has a value between 0.012–0.015 $\pm 0.002$ in dependence of CoFeB composition^58^, which is very close to our value. The bulk CoFeB value of damping can be as low as 0.004^59^, which is over two times smaller than values in our study for CoFeB with 0.8, 0.9 and 1.0 nm thicknesses. Many works are also available for exhibiting low damping parameters accompanied with PMA for CoFeB such as a damping parameter of 0.015 in W/CoFeB/MgO films^49^, 0.014 in different compositions of CoFeB^58^ and ~ 0.01^60^. This implies that within the margin of error, our findings closely approximate or align well with these results. This comparison indicates that low spin pumping effects in Au/CoFeB/Au and Ta/CoFeB/MgO may be responsible for relatively low effective damping. However, we have to remember that also other effects may contribute to the effective damping measured, as it is explained in Ref.^41^.

The effectiveness of a material in magnonics is determined by factors such as the magnon dispersion relation, magnetic anisotropy, damping, and the ability to control and manipulate SWs^59^. So, long propagation distances are required to send coherently the signal codded in SWs, to perform logic operation and read the output signal after that. Our research indicates that the materials with PMA may also be suitable for magnonics, especially that in the out-of-plane orientation of the magnetization the propagation of SWs is isotropic^58^. Also, for the exploitation of magnon–phonon, magnon–photon or magnon–magnon couplings, recently explored for quantum magnonics, the narrow linewidth is a crucial parameter to rich strong-coupling regime^28,29^. From the spintronic point of view, the materials with low damping are promising for high-speed devices as damping relates to the time of the magnetization switching, so its minimizing can help to optimize spintronic devices based on STT or MTJ^61,62^, and also to make these spintronic devices more energetically effective^42^.

## Conclusion

In summary, we successfully investigated the SW dynamics in ultrathin Au/CoFeB/Au films. Most notably, to our knowledge, this is the first study to investigate the SW analysis of this particular composition which confers asymmetry in dispersion relation. It confirms the existence of DMI here and we characterized the values of DMI in the magnetized thin films with strong PMA. Through our investigation, we show that Au/CoFeB/Au is a unique system, offering PMA, DMI and relatively low damping. Our study provides the framework for future studies towards exploitation ultrathin Au/CoFeB/Au multilayers for magnonic applications.

## Data Availability

The datasets used and/or analysed during the current study are available from the corresponding author on reasonable request.
